# Use of rivaroxaban in an elderly patient with intermediate-low early mortality risk due to pulmonary embolism: a case report

**DOI:** 10.1186/s13256-015-0758-6

**Published:** 2015-11-26

**Authors:** Maurizio Menichetti, Sebastiano Rosso, Elisa Menegatti, Maria Pazzaglia

**Affiliations:** UO PS/MURG OC S. Maria delle Croci, Viale Randi 5, 48121 Ravenna, Italy

**Keywords:** Anticoagulant, Case report, Elderly, Mortality risk, Pulmonary embolism, Rivaroxaban

## Abstract

**Introduction:**

Pulmonary embolism remains one of the leading causes of cardiovascular mortality. The standard treatment for pulmonary embolism is anticoagulant therapy using low molecular weight heparin, fondaparinux and a vitamin K antagonist, but a recent clinical trial showed that rivaroxaban, an oral factor Xa inhibitor, was as effective as standard therapy for the initial and long-term treatment of pulmonary embolism and had less bleeding complications.

**Case presentation:**

The present report describes the case of an 80-year-old white man with an intermediate to low early mortality risk of pulmonary embolism. He was successfully treated with rivaroxaban (administered orally as monotherapy), demonstrating rapid benefit without any adverse events.

**Conclusion:**

Rivaroxaban, particularly in the acute phase of pulmonary embolism, may be considered an effective and safe therapeutic choice even in elderly patients, a population less represented in clinical trials.

## Introduction

The elements that constitute venous thromboembolism (VTE) are deep vein thrombosis (DVT) and pulmonary embolism (PE). VTE is the third most frequent cardiovascular disease, with an overall annual prevalence of 100 to 200 cases per 100,000 inhabitants [1, 2].

PE is defined as the blockage of the main pulmonary artery or its branches by substances that have travelled through the bloodstream, most commonly resulting from DVT. It is difficult to assess its epidemiology because it can remain asymptomatic for a long period of time, or its diagnosis may be an incidental finding [2]. PE is responsible for the mortality, morbidity, and hospitalization in a significant number of patients in Europe [3]. The main clinical characteristics of patients with suspected PE are dyspnea, chest pain, fever, hemoptysis, syncope and signs of DVT. In some cases, the first presentation of PE may be sudden death [3, 4]. Diagnosis is based on clinical findings in combination with laboratory tests (such as the D-dimer test) and imaging studies, usually a computed tomographic (CT) pulmonary angiography.

The standard therapy for patients with acute PE is low molecular weight heparin (LMWH), fondaparinux overlapped with and then followed by vitamin K antagonists (VKA); the VKAs are dose-adjusted to keep an international normalized ratio (INR) between 2.0 and 3.0. This regimen is effective but complex. In the EINSTEIN PE clinical trial rivaroxaban, a direct and reversible inhibitor of factor Xa that can be administered orally, demonstrated non-inferiority versus standard treatment in terms of primary outcome (recurrent VTE) in patients with symptomatic PE with objective confirmation, with or without symptomatic DVT. Furthermore, major bleeding was reduced in the rivaroxaban group compared to the standard therapy group [5].

Based on these promising results, we decided to treat an elderly patient with PE without hemodynamic compromise with rivaroxaban.

## Case presentation

An 80-year-old white man was admitted to our emergency department (DEA) presenting with dyspnea; this symptom appeared 2 weeks before and worsened a few days prior to hospital admission. He did not present other symptoms or signs; in particular he did not report thoracic pain or unilateral extremity swelling. He had no previous episodes of VTE, major trauma, immobilization or any other risk factors, such as obesity, cancer or family history suggestive of inherited thrombophilia.

**Fig. 1:**
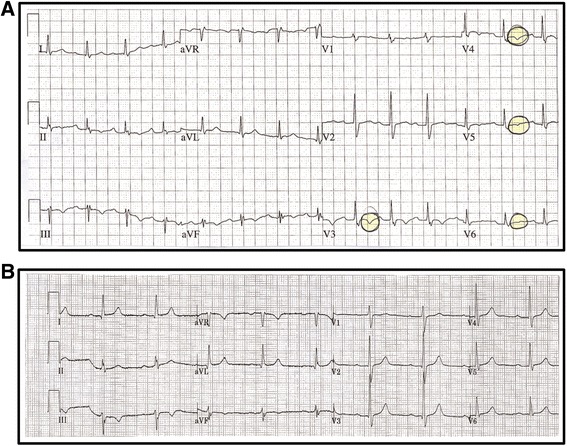
Electrocardiogram on admission and previous electrocardiogram without pathological features. **a** Electrocardiogram on admission: sinus rhythm with inversion of T waves in anterior and lateral leads. **b** Previous electrocardiogram without pathological features

His main predisposing factor for VTE was advanced age. His medical history included arterial hypertension, previous left varicectomy and right saphenectomy. On examination, his systolic blood pressure was 130/80 mmHg and pulse rate was 100 beats per minute. His respiratory rate was 24 breaths/minute and arterial oxyhemoglobin saturation was 90 % at room air. His oral temperature was 36 °C and his mental status was preserved. Chest auscultation did not reveal pathological lung sounds. An electrocardiogram (ECG) showed a normal sinus rhythm with inversion of T waves in leads V3 to V6 (Fig. 1a) not detected in previous recordings (Fig. 1b). Arterial blood gas analysis revealed a mild hypoxemia with respiratory alkalosis. His chest X-ray was normal. No supplemental oxygen was administered.

Laboratory findings on admission revealed a high serum D-dimer level (6987 ng/L), elevated high-sensitivity cardiac troponin concentration (137 ng/l) and increased serum level of pro-B-type natriuretic peptide (pro-BNP; 6166 ng/L), while renal (creatinine 1.53 mg/dl and glomerular filtration rate 43 ml/minute/1.73m^2^) and liver function were normal. The focused assessment with sonography for trauma (FAST) scan performed in DEA showed a moderate right ventricular (RV) dilation and hypokinesia with flattening of his interventricular septum and dilated inferior vena cava.

**Fig. 2:**
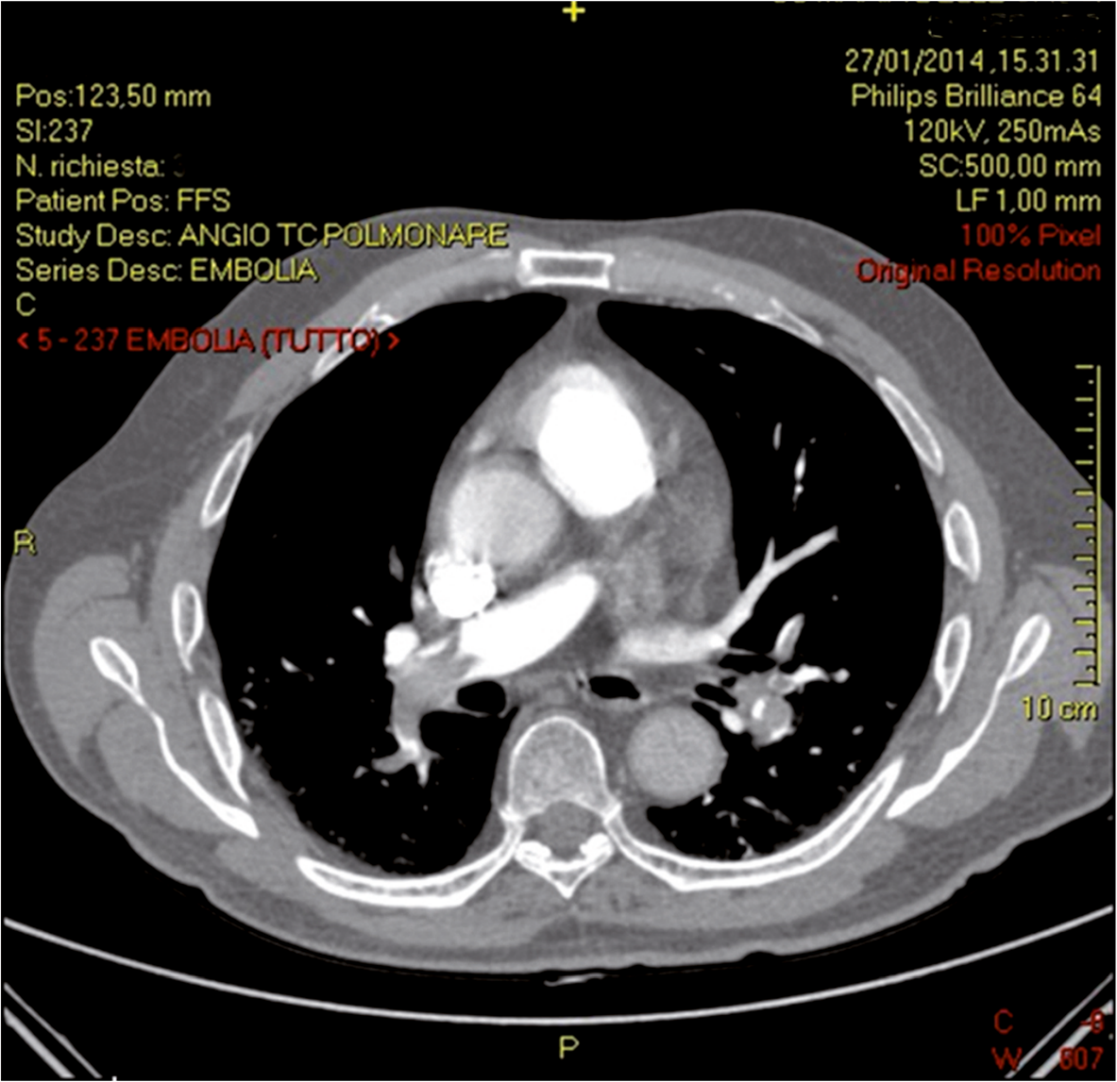
Computed tomographic pulmonary angiography on admission: multiple filling defects within the pulmonary trunk and the right pulmonary artery. Left pulmonary artery presents similar features. These computed tomography findings are suggestive of pulmonary thromboembolism (Miller index >17)

Together, these findings led to the suspicion of PE, subsequently confirmed by a CT pulmonary angiography (Fig. 2).

According to the simplified PE Severity Index (sPESI; Table 1), an extensively validated prognostic tool for predicting death and adverse outcome events in patients with PE, our patient had a score of 0. This data, in combination with the hemodynamic status of our patient, signs of RV dysfunction and cardiac laboratory biomarkers, allowed us to classify our patient as having an intermediate to low risk of early mortality.

**Table 1:** Simplified Pulmonary Embolism Severity Index score

Variable	Simplified Pulmonary Embolism Severity Index score
Age >80 years	1
History of cancer	1
Chronic cardiopulmonary disease*	1
Pulse ≥110 beats/minute	1
SBP <100 mmHg	1
Arterial oxyhemoglobin saturation level <90 %	1

*SBP* systolic blood pressure. *Combined variable of history of heart failure and history of chronic lung disease

Given the diagnosis of bilateral PE and encouraged by the results of the EINSTEIN PE trial [5], we decided to start therapy with rivaroxaban (15 mg, twice daily). In the following days the patient showed a progressive improvement of clinical status and laboratory tests. His troponin levels decreased from 905 ng/L on day 2 to 26 ng/L on day 4, pro-BNP from 6166 ng/L on day 1 to 543 ng/L on day 4 and D-dimer from 6987 ng/L to 1385 ng/L (Table 2). Furthermore, his partial pressure of oxygen (pO_2_) increased from 68.9 to 136.4 mmHg and the following parameters remained in the normal range; pH, partial pressure of carbon dioxide (pCO^2^) and fraction of inspired oxygen (FiO^2^). Supplemental oxygen was not administered.

**Table 2:** Laboratory findings on admission (27 January 2014) and the next 3 days

Day	1	2		3		4
Time (hour)	16:00	08:00	18:00	08:00	18:00	08:00
Troponin T >50 ng/L	137 ng/L	90.5 ng/L	67 ng/L	69 ng/L	60 ng/L	26 ng/L
Pro-BNP <1800 ng/L	6166 ng/L	5495 ng/L	3180 ng/L	1496 ng/L	926 ng/L	543 ng/L
D-dimer <100 ng/L	6987 ng/L	5688 ng/L	3224 ng/L	NA	NA	1385 ng/L

After admission, the patient started treatment with rivaroxaban (15 mg twice a day). *NA* data not available, *Pro-BNP* pro-B-type natriuretic peptide

Five days after starting therapy, we performed another echocardiography showing a normalized RV function: ejection fraction (EF) of 55 %, systolic pulmonary artery pressure (PAPs) of 30 mmHg, and tricuspid annular plane systolic excursion (TAPSE) of 26 mm.

**Fig. 3:**
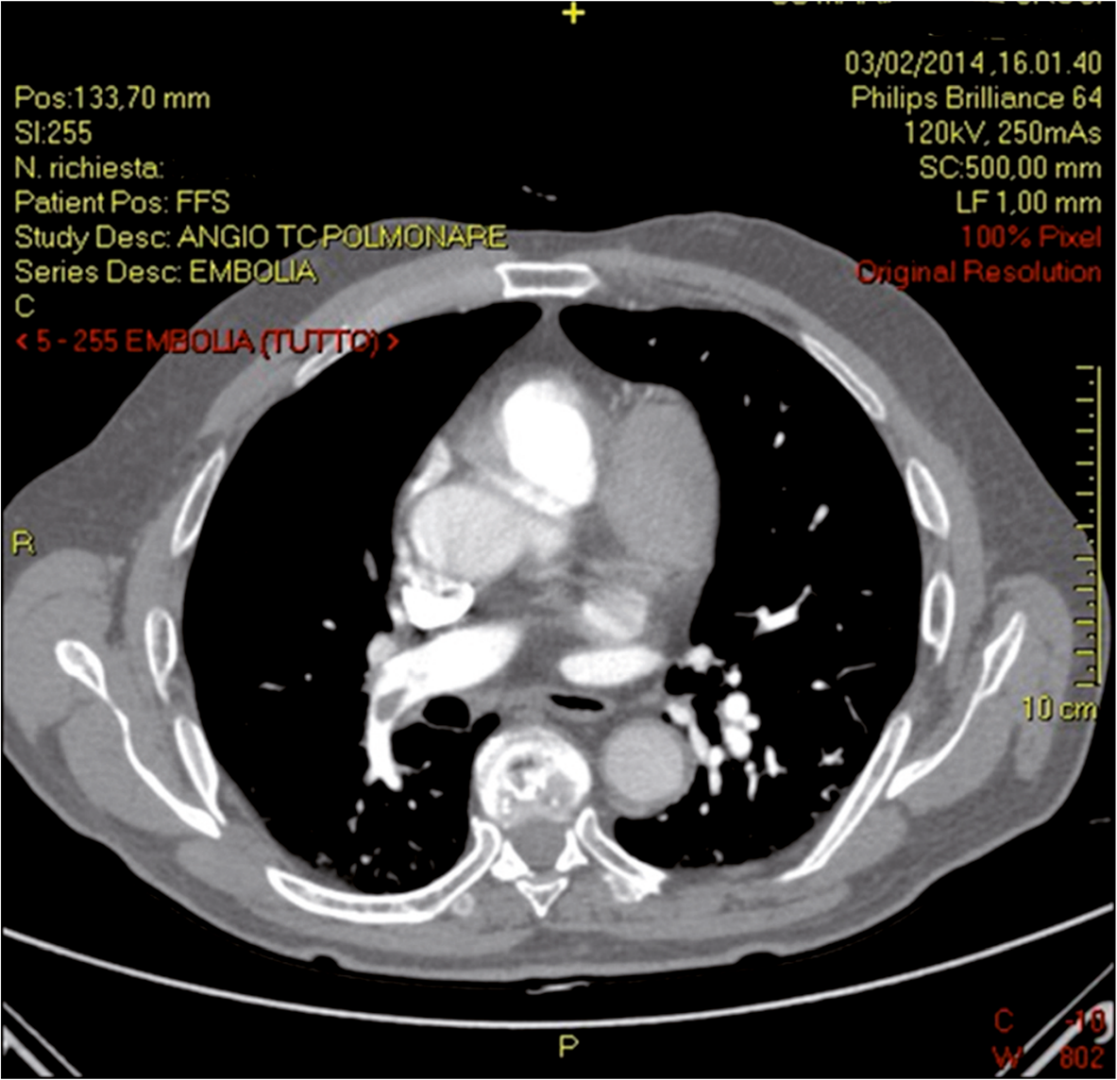
Computed tomographic pulmonary angiography at the 7-day follow-up: partial reperfusion of lower lobe pulmonary arteries. The other computed tomography findings appear unchanged

Seven days after starting treatment, the patient underwent a CT pulmonary angiography that revealed a significant improvement of filling defects in the lower lobes (Fig. 3).

## Discussion

PE is a potentially life-threatening medical emergency that requires urgent intervention. Occlusion of the pulmonary arterial bed can lead to a potentially reversible RV failure. The diagnosis of PE can be missed because of nonspecific clinical presentation. However, early diagnosis is fundamental, since immediate treatment is highly effective [1, 2].

There is an extensive range of patient-related and setting-related risk factors. PE can be ‘provoked’ in the presence of temporary or reversible risk factors (such as surgery, trauma and immobilization) and ‘unprovoked’ in their absence. PE may also occur in the absence of any known risk factor. Patients older than 40 years are at increased risk compared with younger patients and the risk approximately doubles with each subsequent decade [6].

In the present case we describe a patient who presented with dyspnea without any other symptom or risk factor for PE, except that of advanced age. A physical examination and investigations did not support the possible existence of any other cause of dyspnea and led to the suspicion of PE. According to current guidelines [6], given the hemodynamic stability and low PE clinical probability of the patient, we first assessed serum D-dimer levels and then we performed a CT pulmonary angiography. This imaging test supported the diagnosis of bilateral PE. Echocardiography showed a RV dysfunction with significant overload. The combination of clinical findings with imaging and laboratory tests allows stratification of the early mortality risk (understood as in-hospital or 30-day mortality). Our patient, having a sPESI of 0 but both positivity for signs of RV dysfunction and cardiac laboratory biomarkers, was classified as having an intermediate to low early mortality risk.

The sPESI score [7–9] is used by many emergency physicians and acute care internists to stratify the risk of patients with PE in order to determine the most appropriate management approach, including suitability for out-patient care. The score predicts 30-day mortality following PE based on factors such as age, blood pressure, heart rate, oxygen saturation and the presence of chronic cardiopulmonary disease or cancer.

For half a century, the standard therapy for PE has been the administration of parental anticoagulant overlapped with and then followed by a VKA [10]. This regimen has been shown to be extremely effective but with several limitations: requirement for laboratory monitoring and regular dose adjustments, need for injection, narrow therapeutic window and interactions with food and other drugs. Novel oral anticoagulants overcome the limitations of this standard therapy.

Rivaroxaban is a direct and reversible inhibitor of factor Xa that can be administered orally [11, 12]. It offers a simple single-drug approach for initial intensive treatment with a high dose (15 mg twice a day) administered for 3 weeks followed by administration of a standard dose (20 mg once a day). Findings from the recent EINSTEIN PE trial indicate that rivaroxaban was non-inferior compared to standard therapy (2.1 % events in the rivaroxaban group versus 1.8 % in the standard therapy group; hazard ratio 1.12; 95 % confidence interval, CI, 0.75 to 1.68) and no increase in adverse events when compared with standard treatment (LMWH with VKA) [5]. In particular, the safety profile was attractive because major bleeding was observed in 26 patients (1.1 %) in the rivaroxaban group and 52 patients (2.2 %) in the standard therapy group (hazard ratio 0.49; 95 % CI 0.31 to 0.79; *P*=0.003).

This paper presents some similarities with respect to this clinical trial but the age of our patient was not comparable (80 years versus a mean age of 57.9±7.3 years in EINSTEIN PE). Despite the older age of our patient, we felt confident in using rivaroxaban instead of standard therapy. In fact, treatment with rivaroxaban led to rapid improvement and normalization (after 3 days) of PE-related early mortality risk markers.

## Conclusions

In summary, this is the first case report showing that initial intensive treatment with rivaroxaban in an elderly patient resulted in a rapid clinical improvement and normalization of PE-related early mortality risk parameters. Furthermore, no adverse events were noted. This case suggests that rivaroxaban can be considered an effective alternative to standard therapy with LMWH and oral VKA for elderly patients with a hemodynamically stable PE.

## Consent

Written informed consent was obtained from the patient for publication of this case report and any accompanying images. A copy of the written consent is available for review by the Editor-in-Chief of this journal.
